# Supplementary material for: Splice-Junction-Based Mapping of Alternative Isoforms in the Human Proteome
Source: Cell Rep. Author manuscript; Available in PMC 2020 Jan 15. (PMC6961840; doi:10.1016/j.celrep.2019.11.026)
Supplement: 2 [file NIHMS1546469-supplement-2.zip › DF1/PXD010154/Lung/Lung_5_PPP1R35_AEEQPGLGPGAAGR.pdf]

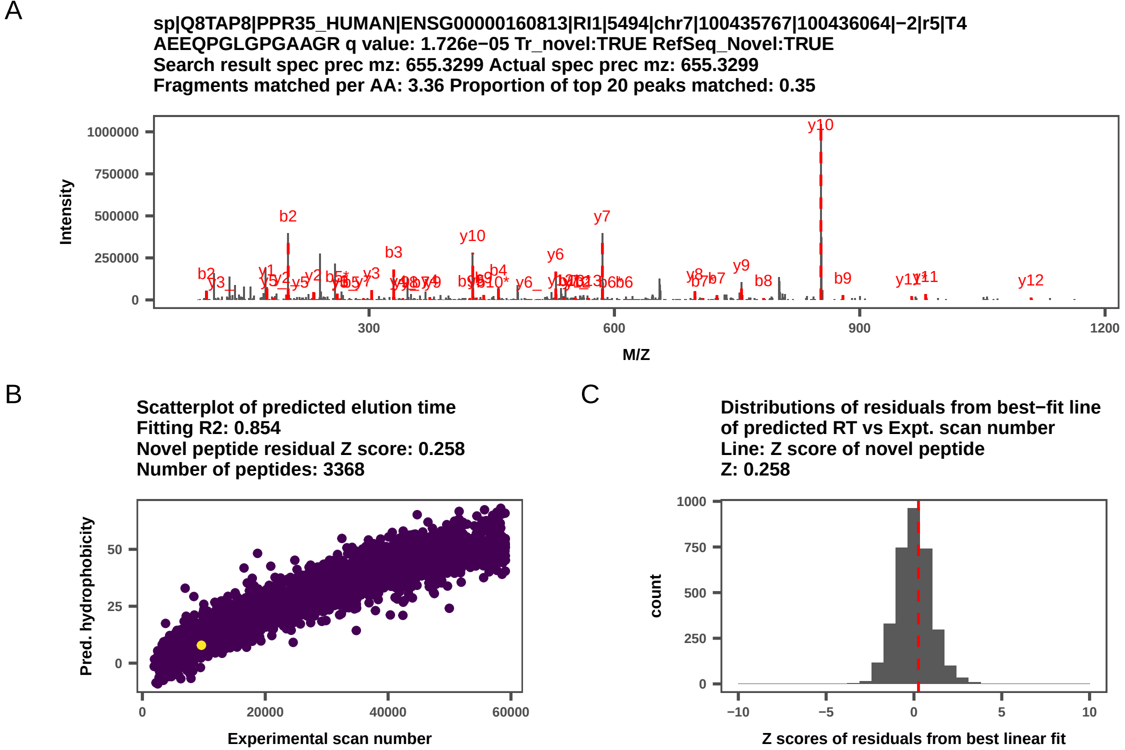
A
sp|Q8TAP8|PPR35_HUMAN|ENSG00000160813|RI1|5494|chr7|100435767|100436064|−2|r5|T4
AEEQPGLGPGAAGR q value: 1.726e−05 Tr_novel:TRUE RefSeq_Novel:TRUE
Search result spec prec mz: 655.3299 Actual spec prec mz: 655.3299
Fragments matched per AA: 3.36 Proportion of top 20 peaks matched: 0.35
Intensity
1000000
750000
500000
250000
0
M/Z
300
600
900
1200
y10
b2
y7
y10
b3
y6
y9
b4
b8
b9
y12
B
Scatterplot of predicted elution time
Fitting R2: 0.854
Novel peptide residual Z score: 0.258
Number of peptides: 3368
Pred. hydrophobicity
50
25
0
Experimental scan number
0
20000
40000
60000
C
Distributions of residuals from best−fit line
of predicted RT vs Expt. scan number
Line: Z score of novel peptide
Z: 0.258
count
1000
750
500
250
0
Z scores of residuals from best linear fit
−10
−5
0
5
10
